# 

**Published:** 1910-06-18

**Authors:** 


### BOOKS RECEIVED.

"Radiumtheraph." Wickman and Degrais. 15s.
"The Nature of Cancer." J. Clay. 3s. 6d.

J. Wright and Sons.

"Errors of Refraction." Chas. Blair, M.D., F.R.C. Second edition.
"Memories." By a Hospital Nurse.
"Diseases of the Colon." By P. Lockhart Mummery.

Smith, Elder and Co.

"Index of Symptoms." R. W. Leftwich, M.D.

Baillière, Tindall and Cox.

"Insanity." By E. G. Younger.
"An Introduction to Hypnotism." By H. E. Wingfield, M.A., M.D.

Williams and Norgate.

"Super Organic Evolution." By Dr. Enrique Lluria.

### Trichinosis.

Œdema with slight erythema over the swollen, tender muscles is very suggestive. In its most characteristic form the œdema occurs in the eyelids and over the eyebrows, and when this appears early in the disease its diagnostic value is considerable. In the hands and feet œdema usually occurs late.

### Camphoric Acid and Night Sweats.

The night sweats of phthisis are probably due to a variety of factors, acting directly or indirectly through the nerve centres. Camphoric acid, given in two doses of 30 grains each during the day, has often been effectual after all other measures have failed to check them. It is more effectual when given in the form of camphoric acid internally than when applied in the form of camphor or camphorated alcohol externally. Camphorated alcohol rubbed into the skin may assist the effects of camphoric acid given internally, but of the two it is the internal remedy that affords by far the greatest benefit.

### Chilblains.

The treatment of this only too familiar trouble is mainly constitutional, and is to be directed to the improvement of the circulation. In addition cold water for washing is to be avoided and tight boots discarded. The wearing of woollen bed socks at night is a useful prophylactic, while the internal administration of calcium chloride seems to have an almost specific effect.

### Scarlet Fever.

Any open wound is extremely liable to become the port of entry to the infecting agent of scarlet fever, and patients after operation should be scrupulously protected from all possible contact with scarlet fever convalescents. That the desquamated epithelium is the principal source of infection is quite erroneous; of far greater importance are discharges from the nose, ears, and throat, while special attention should be paid to the presence of adenoids and to decayed teeth.—*Dr. C. Rolleston.*

THE

### GRADUATES' MEDICAL SCHOOLS.

DIARY FOR THE WEEK, JUNE 20 to JUNE 25.

**ROYAL SOCIETY OF MEDICINE,** 15 Cavendish Square, W. (Temporary address during building.)

At 5 p.m.—**June 22, Special Meeting of Fellows of the Society.**

At Morley Hall, George Street, Hanover Square, W. *Discussion* on "Vaccine Therapy: its administration, value, and limitations." (Sixth meeting.) Re-opened by Dr. W. d'Este Emery, and continued by Dr. W. Camac Wilkinson, Dr. A. C. Inman, Dr. P. Watson Williams, Dr. David Lawson; Sir Almroth Wright will reply.

8.30 p.m.—**June 23, Neurological Section.**

*Pathological Meeting.*—The following papers, illustrated by microscopical specimens and drawings, will be read:—Dr. Walter K. Hunter (Glasgow): "Acute degenerative changes in the nervous system, as illustrated by snake venom poisoning." Dr. H. R. Dean: "Results of the examination of the serum of idiots by the Wassermann reaction." Dr. Farquhar Buzzard and Dr. Hinds Howell: "Secondary growths affecting spinal roots." Dr. Wilfred Harris and Dr. B. H. Spilsbury: "Acute cerebral softening due (?) to venous thrombosis."

**CENTRAL LONDON THROAT AND EAR HOSPITAL,** Gray's Inn Road, W.C.

At 3.45 p.m.—**June 21,** Dr. Atkinson, **Nose.**
3.45 p.m.—**June 24,** Dr. Dan Mackenzie, **Labyrinth.**

**MEDICAL GRADUATES' COLLEGE AND POLYCLINIC,** 22 Chenies Street, W.C.

At 4 p.m.—**June 20,** Dr. Graham Little, **Clinique (Skin).**
5.15 p.m.—**June 20,** Mr. Macleod Yearsley, **The Duty of the General Practitioner towards Deaf Children.**
4 p.m.—**June 21,** Dr. James Collier, **Clinique (Medical).**
5.15 p.m.—**June 21,** Dr. Leonard Guthrie, **Some Nervous Disorders in Childhood.**
4 p.m.—**June 22,** Mr. G. E. Waugh, **Clinique (Surgical).**
5.15 p.m.—**June 22,** Mr. Urban Pritchard, **Meniere's Vertigo.**
4 p.m.—**June 23,** Sir Jonathan Hutchinson, **Museum Demonstration.**
5.15 p.m.—**June 23,** Dr. Wm. Hill, **On the Great Value of the Œsophagoscope in Diagnosis and Treatment** (with an Endoscopic Demonstration on Living Subjects).
4 p.m.—**June 24,** Dr. Dan Mackenzie, **Clinique (Ear, Nose, and Throat).**

**THE POST-GRADUATE COLLEGE,** West London Hospital, Hammersmith, W.

At 10 a.m.—**June 20** and **June 23, Demonstrations by Surgical Registrar.**
12 noon.—**June 20, Pathological Demonstration.**
5 p.m.—**June 20,** Mr. Rickard Lloyd, **Anæsthetics.**
10 a.m.—**June 21, Demonstration of Minor Operations.**
3 p.m.—**June 21** (at Claybury Asylum), Dr. Robert Jones, **Treatment of Varieties of Insanity.**
12.15 p.m.—**June 22,** Dr. Grainger Stewart, **Practical Medicine.**
5 p.m.—**June 22,** Mr. Lloyd Williams, **The Mouth in Health and Disease.**
5 p.m.—**June 23,** Mr. Bidwell, **Intestinal Obstruction.**
10 a.m.—**June 24, Demonstration by Medical Registrar.**
5 p.m.—**June 24,** Dr. Bernstein, **Blood Examination—continued.**

**LONDON SCHOOL OF CLINICAL MEDICINE,** Seamen's Hospital, Greenwich, S.E.

At 2.15 p.m.—**June 21,** Dr. Russell Wells, **The Pulse in Heart Disease.**
3.30 p.m.—**June 22,** Mr. Cargill, **Retrolobular Neuritis.**

**NATIONAL HOSPITAL FOR THE PARALYSED AND EPILEPTIC,** Queen Square, Bloomsbury, W.C.

At 3.30 p.m.—**June 21,** Dr. E. F. Buzzard, **Clinical Cases.**
3.30 p.m.—**June 24,** Mr. Armour, **Surgery of the Nervous System.**

**THE BROMPTON HOSPITAL FOR CONSUMPTION AND DISEASES OF THE CHEST,** Fulham Road, S.W.

At 4 p.m.—**June 22,** Dr. Young, **The Diagnosis and Treatment of Cardiac Irregularities.**

**NORTH-EAST LONDON POST-GRADUATE COLLEGE,** Prince of Wales's General Hospital, Tottenham, N.

At 4.30 p.m.—**June 22,** Dr. A. H. Pirie, **Treatment of Nævus by Radium and Other Methods.**
4.30 p.m.—**June 23,** Dr. A. G. Auld, **Pneumotherapy.**

**ST. MARK'S HOSPITAL FOR DISEASES OF THE RECTUM,** City Road, E.C.

At 2.30 p.m.—**June 24,** Mr. P. L. Mummery, **Colitis.**

**THE HOSPITAL FOR SICK CHILDREN,** Great Ormond Street, W.C.

At 4 p.m.—**June 23,** Dr. Garrod, **Demonstration of Medical Cases in the Wards.**

**Bristol Royal Infirmary.**

### Gifts and Bequests.

Lord Strathcona has sent £200 to University College Hospital in response to the special appeal for £10,000.

Sir Charles Hayes Marriott, M.D., of Kibworth Harcourt, Leicester, left estate of the gross value of £33,545.

Miss Adela Maitland Wilson, of Tunbridge Wells, has left £200 to the Homœopathic Hospital, Tunbridge Wells.

Prince Francis of Teck has received £250 from Mr. Otto Beit, £200 from Mr. and Mrs. F. G. Asher, and £100 from Mr. H. J. King in response to his appeal on behalf of the Middlesex Hospital.

The Skinners' Company has sent £50 to the Westminster Hospital, the fourth instalment of the Company's promised grant of £50 a year for five years.

The Directors both of Parr's Banking Co., Ltd., and Lloyds Banking Co., Ltd., have given generously £105 to the Extension Fund of the Leicester Infirmary.

Mr. Thomas Jones, of Manchester, merchant, has left £100 each to the Manchester Royal Infirmary, the Manchester Royal Eye Hospital, and the Cancer Pavilion and Home, Manchester.

Miss Mary Ann Dugdale Harland, of Ashbourne Lodge, Withington, daughter of the late Dr. Thomas Harland, has bequeathed, subject to life interest, £1,000 to the Salford Royal Hospital.

Mrs. Helen Crowther, of Rochdale, has bequeathed £4,000 to the Rochdale District Nursing Association, £3,000 to the Rochdale Infirmary, £1,000 to the Pendlebury General Hospital and Dispensary for Sick Children.

Mr. Alfred Shipley, of Bristol, has bequeathed £1,500 to the Bristol General Hospital; £500 each to the Bristol Royal Infirmary, the Bristol Children's Hospital, and the Winsley Sanatorium; and £200 to the Bristol Eye Hospital.

The Leicester Infirmary has received legacies of £250 (less legacy duty) and £100 (duty free) under the wills of Mr. John Henry Weston and Mr. Samuel Jaques, of Leicester, and £300 (duty free) for the Children's Hospital under the will of the latter gentleman.

Mrs. Esther Gozina Bernard, of Kentish Town, has bequeathed £200 to the Central St. Pancras (Camden Town) District Nurses' Association, £100 to the Winifred House Invalid Children Convalescent Nursing Home, Tollington Park, and £50 to the New Hospital for Women.

Miss Harriet Sydney Hughes, of Bryn Menai, Bangor, has left her residence, Bryn Menai, Bangor, to the Queen Victoria Jubilee Institute for Nurses as a house of rest or convalescent home for nurses, and a sum not exceeding £6,000 for its upkeep; and £500 to the Carnarvonshire and Anglesey Infirmary.

Mr. John Mitchell, of Bradford, Yorkshire, a governor of the Bradford Royal Infirmary, has bequeathed £300 to the Bradford Royal Infirmary; £50 each to Bradford Eye and Ear Hospital, Bradford Children's Hospital, Woodlands Convalescent Home, Rawdon, Ilkley Hospital, and Craig Hospital, Morecambe.

Miss Mary Alice Richardson, of Barnard Castle, Durham, if at her brother's death there be a cottage hospital in or near Barnard Castle, or the same shall be in the course of erection, left £300 for the building fund thereof.

Mr. Nathaniel Armstrong, of Newcastle-on-Tyne, has left £250 each to the Royal Infirmary, Newcastle, the Northern Counties Orphan Institution, and the Newcastle-upon-Tyne Home for Crippled Children.

Sir William Henry Wilson-Todd, first baronet, of Halnaby Hall, North Riding of Yorks, has left £250 each to the Newcastle-upon-Tyne General Infirmary and the Hull Royal Infirmary.

### Jottings.

The Queen of Norway has allowed the name of Prince Olaf to appear as a patron of the *matinée* at His Majesty's Theatre on July 15 in aid of the Royal Waterloo and Infants Hospitals, and has also sent a donation of £5 5s.

Out of respect to the memory of King Edward VII., who was a patron of the Royal Hospital for Incurables, it has been decided to abandon the festival dinner at the Grocers' Hall. The contributions received and promised up to the present in connection with the festival dinner amount to £4,168.

The Governors of the Princess Alice Hospital, Eastbourne, have decided to make inquiries with a view to erecting a new hospital on a central site, and it was suggested that there could be no more appropriate memorial to the late King.

The first list of donations towards the memorial to King Edward, to consist of a statue and a new children's hospital, was published by the *Birmingham Daily Mail* last week. The total is £5,093. A sum of £1,000 is given by Mr. W. Waters Butler, Mr. H. A. Butler, and Miss Nellie Butler to endow a bed in the children's hospital to the memory of their late father. A similar amount is contributed by Mr. George Cadbury, and £1,221 by Messrs. Mitchell and Butler. Mrs. E. A. Avins, Miss E. A. P. Avins, and Mr. J. A. Kenrick have given £500 each.

As a result of recent improvements at the Infectious Diseases Hospital at Myland, Essex, two new blocks have been opened at a cost of about £2,000. The larger block—named the Laver Block, out of compliment to the Chairman of the Sanitary Committee (Dr. Henry Laver)—has sixteen beds, and the smaller block—the Shaw Block—has four beds. The new buildings were inspected by members of the Colchester Town Council, among whom were the Mayor (Mr. Councillor E. A. Blaxill), Mr. Alderman Laver (Chairman), and Mr. Councillor W. C. Shaw (Vice-Chairman of the Sanitary Committee).

During the past year the Convalescent Homes Association has tried to assist some of its members in obtaining grants from King Edward's Fund. A circular letter was sent to the Homes in May 1909 inquiring if a grant had been obtained from King Edward's Fund. Those Homes affiliated to the Association which replied that they had not been assisted were visited by two members of the Executive Committee, and, in all but two instances, approved. A report was sent to each Home inspected, and a general letter was sent to the Distribution Committee of King Edward's Fund, mentioning the Homes which had been approved. It is hoped that this may influence the Distribution Committee of King Edward's Fund.

### TO HOSPITAL SECRETARIES AND OTHERS.

We invite contributions from any of our readers, and shall be glad to pay, according to length, for items of news for the institutional section (including appointments, resignations, gifts, etc.) which we may publish. All payments for manuscripts used are made as early as possible after the beginning of each quarter.

### Appointments.

Mr. Brooks-Broadhurst and Mr. C. J. Wilkinson, Sebergham Castle, have been appointed to fill two vacancies on the Committee of Election at the Cumberland Infirmary.

Mr. D. E. Derry, M.B., Ch.B. (Edinburgh), has been appointed Assistant in the Department of Anatomy and Curator of the Anatomical Museum at University College for the session 1910-11.

Mr. E. K. Martin, M.B., B.S., F.R.C.S., has been appointed Demonstrator in Anatomy at University College for the session 1910-11.

The Directors of the London, Brighton and South Coast Railway Company have appointed Mr. William Turner, M.S., F.R.C.S., of 17 Harley Street, Medical Officer of the Company in succession to the late Professor William Rose.

### COMING EVENTS OF THE WEEK.

Saturday, June 18.—National Fire Brigade Tournament in aid of the Leicester Infirmary and Children's Hospital, Leicester.

Carnival at Stourton in aid of Leeds Hospital.

Sunday, June 19.—Carnival at Stourton.

Monday, June 20.—Lord Strathcona presides at Festival dinner of the Metropolitan Hospital, Whitehall Rooms, 7.

Meeting of Executive Committee of General Committee of Medical Men and Poor Law, 30 Harley Street, W., 4.30.

Tuesday, June 21.—Royal Hospital, Chelsea, Pulpit State Fund Supper.

Wednesday, June 22.—Harben Lecture, Professor Sir William Leishman, Institute of Public Health, 6.

Thursday, June 23.—Distribution of Prizes, St. Thomas's Hospital, 3.

Charing Cross Hospital Matinée, Adelphi Theatre.

### Medical Training of Women.

The annual meeting of the North India School of Medicine for Christian Women (Ludhiana) was held on Tuesday, June 7, in the Connaught Rooms, Freemasons' Hall, Colonel G. Gordon Young (late a Commissioner in the Punjab) presiding. The school was founded in 1894 by Dr. Edith Brown, in conjunction with a committee composed of medical and educational missionaries and others, who realised the need of providing medical training, combined with the influence of a Christian home, for the Christian women of India. The commencement, said the Chairman, had been so small, and the difficulties so large, that there had seemed big probabilities of failure; but he was glad to say they had to report advances, not reverses, and that the school might be said to have passed out of the experimental stage. There is a well equipped hospital in connection with the school, in which during the past year 24,483 out-patients and 1,349 in-patients had been medically treated, all of whom had come under direct Christian influence and instruction.

Miss L. M. Hill, the Secretary, spoke of the great distances from which the patients came so that they might secure the benefits of the Ludhiana Hospital. All were most grateful, and some showed their gratitude by substantial gifts. For instance, one patient had given enough to build two private wards and to instal the telephone between the doctor's house and the hospital, and another had built a well, while the people of the town had given Rs. 3,000 for the enlargement of the hospital.

Miss Edith Brown, M.D., gave a very interesting account of the work and its necessities. The past year had witnessed a good deal of opposition from the Aryas, who evidently wished to be independent, and under no obligations to the Christians. The *Arya Gazette*, published in Lahore, said in June last: "The most powerful weapon which the Christians have to lay hold of the hearts of the Hindu women is the Zenana Hospital." Such an admission showed how those who bitterly opposed the work had to confess that it was exercising a powerful influence. The medical school had been a centre of great influence. It had trained students who had gone out to distant parts of India to create fresh centres of Christian usefulness; so that if the school were to close its doors to-morrow it would not have existed in vain, as it had already sent its representatives far and wide to care for both the souls and the bodies of the people.

It was explained that the present great needs of the school were two more doctors and two more hospital sisters. Money was also needed for maintenance and extension and salaries, especially for the support of 50 more beds at £10 each per annum, and to provide 20 more scholarships at £15 each. A total of £10,500 was also greatly needed for the extension and proper equipment of the school.

Dr. Chatterjee, a Presbyterian minister stationed near Ludhiana, gave emphatic testimony to the value of the work of the school and hospital. From the former he had received highly trained and most useful workers, who were now assisting him in his labours. He was perfectly convinced that the school was supplying a felt need in connection with medical training for women. He himself, in order to avoid the dangers to which women students are exposed in mixed colleges (men and women), had sent his daughter to America for her medical training before the school at Ludhiana was opened.

